# Factors affecting cardiovascular morbidity in young FMF patients. A comparative analysis in colchicine treated FMF patients with and without cardiovascular disease

**DOI:** 10.1186/1546-0096-13-S1-O47

**Published:** 2015-09-28

**Authors:** AJ Roitman, I Ben Zvi, O Kukuy, A Livneh

**Affiliations:** 1 Sheba Hospital, FMF outpatient clinic, Tel Hashomer, Israel; 2 Sheba Hospital, Institute of Nephrology and Hypertension, Tel Hashomer, Israel

## Background

Familial Mediterranean fever (FMF) is the prototype of chronic auto-inflammatory diseases. During the FMF attacks there is an uncontrolled activation of an inflammatory cascade with a consequent release of many pro-inflammatory molecules, which subsides or slows down in between the attacks. Chronic inflammation has been found to be associated with higher incidence of atherosclerotic cardiovascular disease (CVD). Thus, being a chronic inflammatory disorder, it is speculated that FMF may be considered as an independent risk factor for CVDs. Most studies looking at the association between FMF and CVDs have focused on markers, suggesting increased atherosclerosis in FMF as compared to the general population. Yet, these studies yielded conflicting results. In the present study we analyze atherosclerosis morbidity in FMF, by comparing affected to unaffected FMF patients.

## Objective

To determine FMF related and other underlying factors leading to cardiovascular disease in FMF.

## Methods

All files of colchicine treated FMF patients, 50 years old or less, cared for in Sheba Medical Center (the largest FMF Center in Israel, with registered FMF population larger than 10000 patients), as in patients or FMF clinic outpatients, over the last 10 years, bearing a diagnosis of cerebral, cardiac or peripheral vascular disease were pulled out and reviewed. For each studied patient 2 FMF control subjects were adjusted from patients arriving to FMF clinic for their periodic follow up visit. Our endpoints were: 1. Incidence of FMF related (without additional risk factors) CVD in this population compared to the general population 2. Elucidation of FMF related and unrelated risk factors for CVD in FMF. FMF severity, one of the FMF related factors studied, was assessed using the severity score-2 (SS-2) by Mor et. Al.

## Results

There was only one FMF patient younger than 50 year old, who suffered of CVD and had none of the traditional risk factors. All other FMF patients with CV morbidity (23 cases) had other risk factors for CV disease. Compared with the control FMF subjects, none of the assessed FMF related parameters, including increased disease severity, was more common in FMF-CVD. However, in the FMF-CVD cohort, the rate of other inflammatory diseases was higher.

## Conclusion

These findings suggest that in colchicine treated FMF population younger than 50 years of age, FMF per se is not a risk factor for CVD.

